# Cutaneous Adverse Reactions to SARS-CoV-2 Vaccines: A Systematic Review and Meta-Analysis

**DOI:** 10.3390/vaccines10091475

**Published:** 2022-09-06

**Authors:** Francesco Bellinato, Zeno Fratton, Giampiero Girolomoni, Paolo Gisondi

**Affiliations:** Section of Dermatology and Venereology, Department of Medicine, University of Verona, 37126 Verona, Italy

**Keywords:** SARS-CoV-2, COVID-19, vaccines, cutaneous adverse reaction, systematic review, meta-analysis

## Abstract

**Background:** An increasing number of cutaneous adverse reactions (CARs) to SARS-CoV-2 vaccines have been reported, but their incidence is debated. **Objective:** To estimate the pooled incidence of CARs to SARS-CoV-2 vaccines in the general adult population. **Methods:** A systematic review and meta-analysis of original articles published on MEDLINE via PubMed and Web Of Science from 1 January 2020 to 18 July 2022 was undertaken. Studies reporting the incidence proportion of CARs (defined as number of new cases of CARs on the total of vaccinated people) were included. All types of SARS-CoV-2 vaccine were included. People receiving at least one dose were considered eligible. Local cutaneous reactions were excluded. **Results:** A total of 970 records were identified and screened by title and abstract; 22 observational studies were included with aggregate data on 93,165 participants. The pooled incidence of overall CARs was 5% (95%CI 4–6%; I^2^ = 99%; *p* < 0.001), ranging from <0.01 to 19.00%. Most CARs were new onset dermatitis including rash, urticaria and vascular lesions; one case of Steven–Johnson syndrome and six cases of erythema multiforme were reported. In the sensitivity analysis we found that the incidence of CARs after the first and second dose was similar, i.e., 3% (95%CI 2–3%; I^2^ = 96%; *p* < 0.001) and 3% (95%CI 2–4%; I^2^ = 97%; *p* < 0.001), respectively. The magnitude of incidence of CARs remained unchanged independently of vaccine platform and in the general population versus healthcare workers. **Conclusions:** CARs associated with SARS-CoV-2 vaccines are frequent but mild and self-remitting, whereas severe CARs are rare.

## 1. Introduction

The development of safe and effective vaccines has been an overriding priority for controlling the 2019 coronavirus disease (COVID-19) pandemic. By December 2020, the United Kingdom and the Food and Drug Administration (FDA) immediately issued the emergency use authorization (EUA) for the Pfizer-BioNTech (BNT162b2) vaccine [1]. This was not only the first mRNA vaccine approved for human use but also the fastest formulated vaccine whose development was initiated just 11 months back (January 10, 2020), without long-term safety data [2]. Then, the FDA issued the EUA for another mRNA vaccine, i.e., ‘mRNA-1273’, also known as the ‘Moderna COVID-19 vaccine’. BNT162b2, mRNA-1273 and ChAdOx1 nCoV-19 by Astra Zeneca marketing authorization in Europe was issued some weeks later by the European Medical Agency [3]. Since then, several SARS-CoV-2 vaccines have been authorized and approved for distribution around the world, with many more in the pipeline [3]. As a massive SARS-CoV-2 vaccination campaign is underway, increasing reports of adverse events associated with the vaccines have emerged. Common side effects are mild and include dizziness, headache, pain, muscle spasms, myalgia and paresthesia. In rare cases, serious adverse events including thrombosis, stroke, neurological adverse events (i.e., Guillain Barrè Syndrome, transverse myelitis, and acute disseminated encephalomyelitis) and myocarditis have been reported [4]. An increasing number of cutaneous adverse reactions (CARs) associated with the SARS-CoV-2 vaccination has been also described, but their incidence remains debated. The objective of this study is to estimate the pooled incidence of CARs associated with SARS-CoV-2 vaccination in the general adult population.

## 2. Methods

### 2.1. Registration of the Protocol

The protocol of this systematic review and meta-analysis was registered in PROSPERO with the number CRD42021265351.

### 2.2. Search Strategy

This study was performed according to the Preferred Reporting Items for Systematic Review and Meta-Analysis (PRISMA) and Meta-analysis Of Observational Studies in Epidemiology (MOOSE) guidelines [5,6]. We conducted an extensive search in MEDLINE via PubMed and Web of Science for original articles published from 1 January 2020 to 18 July 2022. No other additional sources were consulted. The search strategy included a combination of free text key terms (Supplementary Table S1). No restrictions in terms of sex, race, or geographic area were applied. MeSH terms were not used since this research topic is recent and new studies might not be indexed with MeSH terms at the time of writing. References of relevant original papers and review articles were also screened for other eligible studies not included in the database primary search.

### 2.3. Study Selection Criteria

Original articles reporting the incidence proportion of CARs (defined as number of new cases of CARs on the total of vaccinated people) in the general adult population and healthcare workers were included. All types of SARS-CoV-2 vaccine were considered (i.e., viral vector, mRNA, inactivated and protein-based). People receiving at least one dose were considered eligible. Local cutaneous reactions to vaccines were excluded. Articles in languages other than English, reviews, expert opinions, position statements, book chapters, posters, abstracts, meta-analysis, commentaries, and articles reporting pre-authorization studies were also excluded.

### 2.4. Data Extraction

After duplicate removal of the primary search results, the title and abstract of the retrieved articles were independently reviewed by two authors (FB and ZF). Then, selected articles underwent full-text evaluation for eligibility and data extraction. A more experienced researcher (PG) was consulted in case of discrepancy between authors. The following data were extracted, i.e., author, publication year, study design, study time frame, study population, vaccine type, CARs overall incidence, CARs incidence after the first and second dose, CARs incidence after both doses, CARs incidence per vaccine type and phenotype of CAR. Phenotypes of CARs were reported according to the diagnosis provided by the authors and were classified as new onset skin reactions or flares of pre-existing dermatoses [7]. The extracted data were collected and managed on a Microsoft Excel spreadsheet. No author was contacted in case of missing data.

### 2.5. Risk of Bias Assessment

Two authors (FB and ZF) independently assessed the risk of bias of the cross-sectional studies included in the quantitative analysis based on the Johanna Briggs Institute (JBI) critical appraisal checklist for studies reporting prevalence data [8,9]. The items included: (1) appropriateness of the sample frame, (2) appropriateness of population sampling, (3) adequateness of the sample size, (4) level of detail of study subjects’ description, (5) coverage bias, (6) validity of the outcome measurement instrument, (7) reliability of the outcome measurement, (8) appropriateness of the statistical analysis, (9) adequateness of the response rate. For each item one of the following assessments was given: yes, no, unclear, not applicable.

### 2.6. Statistical Analysis and Synthesis

Pooled incidence and 95% confidence intervals (95%CI) were used to summarize the weighted effect size for each study using the DerSimonian–Laid random-effects model. Confidence intervals were computed using the exact binomial method. Statistical heterogeneity was calculated using the *I*^2^-statistics, which provides an estimate of the percentage of variability across studies that is due to heterogeneity rather than chance alone. According to Higgins and Thompson, *I*^2^-values of approximately 25% represent low heterogeneity; approximately 50% represent medium heterogeneity; and approximately 75% represent high heterogeneity. To explore the possible sources of heterogeneity among the eligible studies we performed subgroup analyses stratifying the eligible studies by vaccine type, study country and study population. As a sensitivity analysis, the incidence of CARs after the first and second single doses were pooled when reported.

Funnel plots analysis was performed to detect publication bias [10]. For all statistical tests, a significance level of *p* < 0.05 was considered. We used Review Manager version 5.3 (Copenhagen: The Nordic Cochrane Centre, The Cochrane Collaboration, 2014) and STATA^®^ software v16.1 (StataCorp, College Station, TX, USA) for all statistical analyses.

## 3. Results

### 3.1. Characteristics of the Included Articles

**Figure 1:**
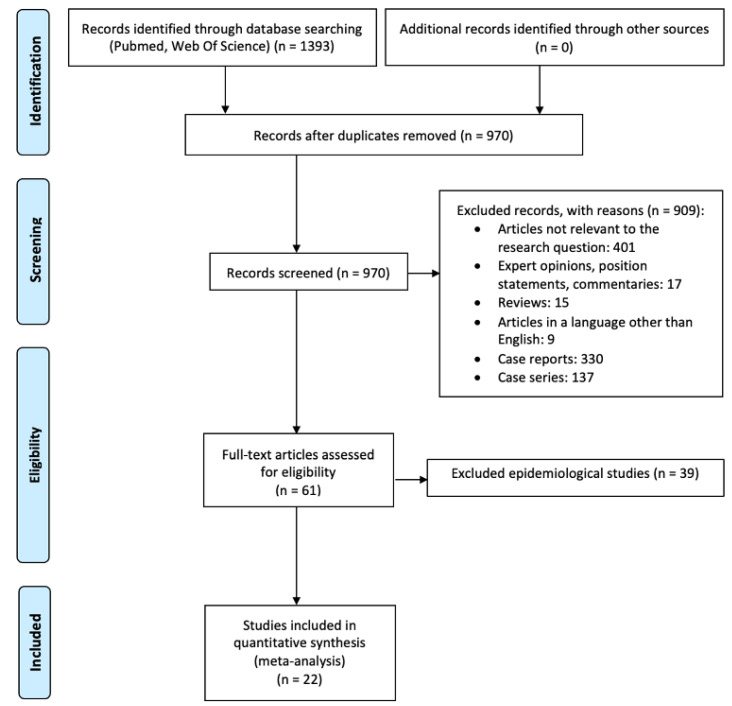
PRISMA Study Flow Chart describing the screening procedure of the articles included in the study.

The PRISMA study flow chart describing the screening procedure of the articles included in the study is reported in Figure 1. A total of 1393 articles were retrieved by literature research. Of these, 423 duplicates were identified and removed. A total of 970 articles went through title and abstract screening. Of those, 909 were excluded because they did not meet the inclusion criteria. The remaining 61 articles were full-text screened. Among these, 39 studies were excluded based on the eligibility criteria (Supplementary Table S2). A total of 22 cross-sectional studies [11,12,13,14,15,16,17,18,19,20,21,22,23,24,25,26,27,28,29,30,31,32] (including 21 surveys and one registry-based study) with aggregate data on 93,165 individuals were included. The characteristics of each study selected are summarized in Table 1 and Supplementary Table S3.

Most of the studies were conducted in Asia and Europe. In 14 studies a female predominance was found, in 6 studies gender distribution was not reported. The vaccine platforms encompassed Pfizer-BioNTech, Moderna, Covishield-Astra Zeneca, Sinopharm, Sputnik, Bharat, Cuba-Pasteur and CoronaVac. Most studies considered RNA vaccines or included together reactions following different vaccine platforms. Nine out of 22 studies specified CARs incidence after the first and second doses. In 16 out of 22 studies the sample consisted of healthcare workers. Relevant limitations of some of these studies were the unstandardized method of identification of CARs, being often self-reported and captured through online surveys and/or questionnaires. CARs were often not exhaustively detailed and described generically as itchy skin rash or urticaria.

### 3.2. Incidence of Cutaneous Adverse Reactions

**Figure 2:**
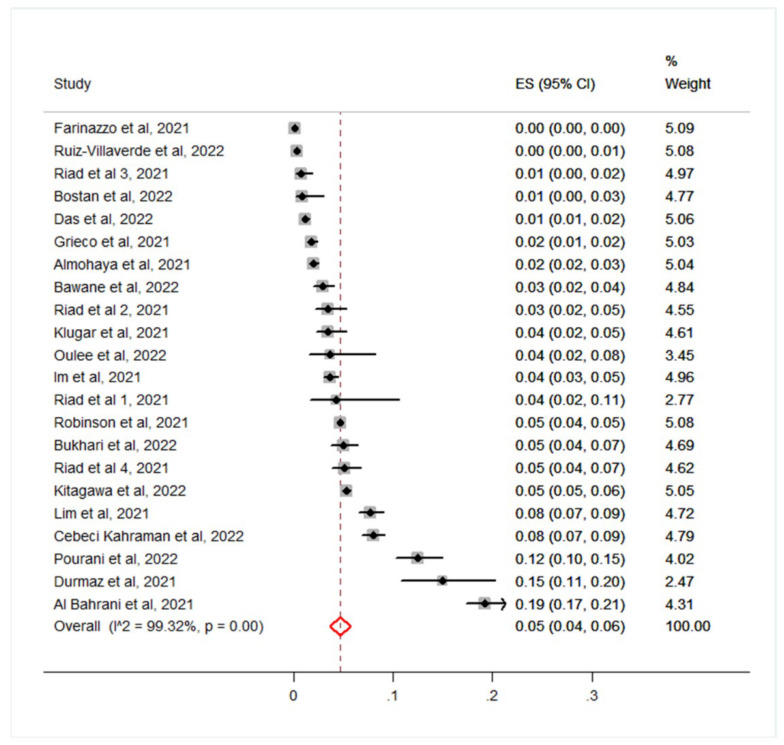
Forest plot and pooled estimates of the incidence of cutaneous adverse reaction to SARS-CoV-2 vaccines in 22 eligible studies.

The pooled incidence of CARs was 5% (95%CI 4–6%; I^2^ = 99%; *p* < 0.001), ranging from <0.01% to 19.00% (Figure 2). Most CARs were new-onset skin reactions. In particular, exanthema generally described as an itchy rash (including morbilliform and pityriasis rosea-like eruption), and urticaria were the most commonly reported. Petechial rash was observed in 18 and cutaneous small vessel vasculitis in three patients, respectively. Other rarer CARs included lichenoid and eczematous lesions, and autoimmune bullous disorders (including bullous pemphigoid, pemphigus vulgaris and pemphigus foliaceus). Regarding severe CARs, Steven-Johnson syndrome and erythema multiforme were reported in one and three patients, respectively. Exacerbations of chronic cutaneous dermatoses such as psoriasis, and cutaneous lupus erythematosus were more rarely reported (Supplementary Table S3).

Subgroup analysis was performed based on vaccine type, study country and study population (i.e., general population vs. healthcare workers) to assess sources of heterogeneity (Supplementary Figures S3–S5). Stratifying by vaccine type the incidence of CARs ranged from 3% (95%CI 2–5%) to 5% (95%CI 3–7%) in studies evaluating only RNA vaccines and those including together different vaccine platforms, respectively. In a single study assessing reactions to the inactivated vaccine the incidence was 15% (95%CI 11–20%) [18]. Stratifying by study country, the incidence of CARs varied from 2% (95%CI 1–2%), to 5% (95%CI 4–5%), to 7% (95%CI 5–8%) in European, US and Asian studies, respectively. Pooled incidence from studies performed on healthcare workers showed almost no differences with respect to the general population, i.e., 4% (95%CI 3–5%) vs. 6% (95%CI 3–9%), respectively. In subgroup analyses, the high degree of heterogeneity remained essentially unchanged. The Funnel plot and Egger test revealed publication bias (*p* < 0.001) (Supplementary Figure S6).

**Figure 3:**
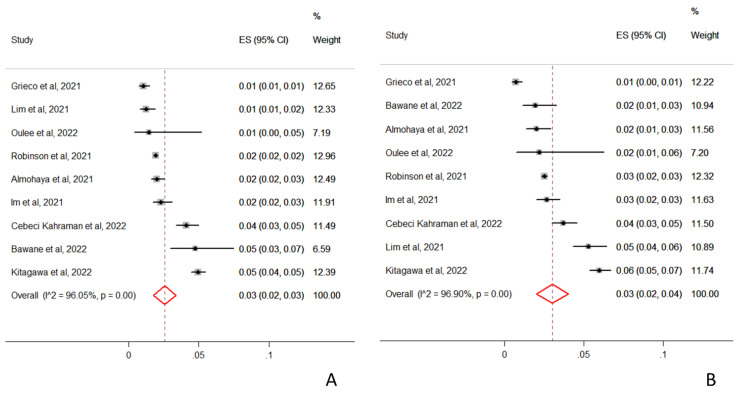
Forest plot and pooled estimates of the incidence of cutaneous adverse reaction to first (**A**) and second (**B**) dose of SARS-CoV-2 vaccines in 9 eligible studies.

A sensitivity analysis performed on the pooled incidence of CARs following the first and the second vaccine dose based on the data of nine studies that reported this data revealed that the incidence of CARs following the first and the second vaccine dose was very similar, 3% (95%CI 2–3%; I^2^ = 96%; *p* < 0.001) and 3% (95%CI 2–4%; I^2^ = 97%; *p* < 0.001), respectively (Figure 3A,B) [12,13,16,20,21,22,24,25,31].

### 3.3. Risk of Bias

Results of the risk of bias assessment are summarized in Supplementary Figures S1 and S2 and Supplementary Table S3. In most studies, the sample frame was not properly suitable to address the target population as not derived from registries or large cohorts. Diagnosis of CARs was generally self-reported and captured through online surveys and/or questionnaires. Most studies presented an uneven gender distribution, with a female predominance. Most studies did not report the sample size estimation. Most studies did not report information about individuals’ medical history. Some studies reported the presence of dropouts but did not provide any details about their characteristics. In almost all studies the statistical analysis was appropriate.

## 4. Discussion

The main finding of the study is that CARs to SARS-CoV-2 vaccines are frequent (>1/1.000–<1/100), with an overall pooled incidence of 5% (95%CI, 4–6%) [33]. Nonetheless, CARs are less common when compared to local skin reactions (i.e., pain, redness, and swelling at the vaccination site) and systemic adverse events (i.e., fever, fatigue, headache, chill, vomiting, diarrhea, nausea, and arthralgia). In a systematic review and meta-analysis by Sharif N et al., pain at the injection site was the most common local symptom in the mRNA and adenovirus vector vaccines, affecting up to 85% and 78% of the patients, respectively [34]. Fever, headache and fatigue were the most commonly reported systemic symptoms, affecting up to 95%, 68% and 55% of the patients [34]. Our finding is consistent with the recent systematic review and meta-analysis by Washrawirul C. et al. [35] who found an incidence of 5.9% (95%CI 3.8–8.8%) [35]. We also found that the incidence of CARs after each dose of vaccine is similar. This finding is also consistent with the finding of Washrawirul C. et al. which reported a pooled incidence of 4.2% after the first and 4.0% after the second dose [35]. In fact, recurrence after booster inoculation can occur in those patients who experienced a reaction after the first dose, but also CARs may develop among those with no CARs after the first dose [31]. Most CARs were new-onset skin reactions including rush and urticaria with a benign self-remitting clinical course, whereas severe CARs such as Steven–Johnson syndrome or erythema multiforme were more rarely reported. Similarly, exacerbations of chronic cutaneous dermatoses such as psoriasis, and cutaneous lupus erythematosus were more rarely reported.

Interestingly, we found a slightly higher incidence of CAR in Asian compared to European studies, for which we do not know how to give a precise reason other than to presume it is linked to more careful reporting. We did not find an increased incidence of CARs associated with any selected vaccine platform, but we acknowledge that multiple vaccine types were included in the same studies. Finally, the incidence of CARs among healthcare workers was similar to the general population. However, we acknowledge that 16 out of 22 studies included in the meta-analysis were conducted on healthcare workers. All studies had an uneven gender distribution, with most studies having higher percentages of females than males.

Besides vaccines for COVID-19, other anti-infective vaccinations have been studied and their CARs characterized. Some of them, such as hepatitis B and bacillus Calmette-Guerin vaccines, can be associated with CARs, even if more rarely when compared to COVID-19 [36]. Influenza, varicella, diphtheria/tetanus/pertussis, measles, poliomyelitis, rubella, pneumococcus, tick-borne encephalitis, smallpox, meningococcus and influenza vaccines are even less frequently encountered [36,37]. Different patho-mechanisms may be involved in the development of non-local CARs to vaccination, reflecting the wide heterogeneity of these reactions [37,38]. Particularly following COVID-19 vaccination these might include classical Th1 and Th2 polarized inflammatory reactions, innate immune system activation with Th17/Th22-polarization and macrophages/histiocytes and granulomatous reactions [35,39].

This review is burdened by some limitations. We found a high degree of heterogeneity between the included studies. This could be related to different follow-up times, population characteristics and interindividual variability in the CARs detection. In particular, the incidence of CARs was estimated mostly from surveys/questionnaires. Self-reporting confirmed by a dermatologist was therefore the main instrument used to measure the incidence of CARs. Some studies did not report dropouts following the first dose, and it cannot be excluded that some participants decided not to receive the second vaccine dose after they had developed an adverse reaction following the first one. Not every study reported data on medical history and history of COVID-19 infections among participants. We excluded the randomized controlled trials from the meta-analysis because such studies focused only on local CARs. Including studies assessing only local CARs might negatively underestimate the effect size and further increase heterogeneity. Moreover, findings from observational studies might better reproduce what can generally be seen in clinical practice. We did not investigate CARs incidence in selected patient subpopulations such as those receiving immunosuppressive drugs that may affect the risk of CARs. Of note, the registries mainly refer to younger patients; the elderly, whose vaccination was prioritized in certain countries, are underrepresented. An important caveat is the high risk of bias of the studies included in the meta-analysis, particularly in the validity and reliability of the outcome measurement. Our metanalysis did not include other studies that were indexed after we performed the research strategy, such as Freeman EE et al [40]. Finally, it was not possible to estimate the incidence of rarer and of more significant clinical importance cutaneous manifestations, which would mainly be extracted by analysis of case reports.

In conclusion, CARs associated with SARS-CoV-2 vaccines are frequent but mild and self-remitting, whereas severe CARs are rare. Additional studies conducted with rigorous methodology may provide more reliable estimates of the incidence of CARs in the general population and in specific subgroups of patients.

## Figures and Tables

**Table 1:** Characteristics of the studies included in the metanalysis.

First Author	Study Population	Sample Size	Country	Age, Mean (SD)	Female (%)	Time-Frame	Vaccine Producer	General Incidence of CARs (%)
Al Bahrani et al.	General population	1592	Saudi Arabia	37.4 (9.6)	19	10 April–20 May 2021	Astra Zeneca	NR
Almohaya et al.	General population	3639	Saudi Arabia	37.0 (28.0–48.0) *	63.3	29 May–8 June 2021	Pfizer-BioNTech	73/3639 (2.00%)
Bawane et al.	Healthcare workers	1029	India	NR	NR	16 January–16 August 2021	Covishield-Astra Zeneca, Covaxin	30/1029 (2.92%)
Bostan et al.	Healthcare workers	234	Turkey	31.51 (9.25)	67.1	NR	CoronaVac, Pfizer-BioNTech	2/234 (0.85%)
Bukhari et al.	General population	1021	Saudi Arabia	NR	70.7	1 June–30 September 2021	Astra Zeneca, Pfizer-BioNTech	51/1021 (5.00%)
Cebeci Kahraman et al.	General population	2189	Turkey	50,4 (17.9)	56.4	15 April–15 July 2021	CoronaVac, Pfizer-BioNTech	175/2189 (7.99%)
Das et al.	General population	4063	India	36.7 (19–86) ^	37	September–November 2021	Covishield-Astra Zeneca	50/4063 (1.23%)
Durmaz et al.	Healthcare workers	221	Turkey	Male: 37.03 (13.83)Female: 38.56 (13.29)	50.2	January–March 2021	CoronaVac	NR
Farinazzo et al.	Healthcare workers	19485	Italy	NR	NR	January 2021	Pfizer-BioNTech	28/19485 (0.14%)
Grieco et al.	Healthcare workers	2740	Italy	NR	NR	January–July 2021	Moderna, Pfizer-BioNTech, Astra Zeneca	50/2740 (1.82%)
Im et al.	Healthcare workers	2498	South Korea	NR	NR	March–April 2021	Pfizer-BioNTech	93/2498 (3.72%)
Kitagawa et al.	Healthcare workers	12,109	Japan	NR	§	15–19 July and 19–22 August 2021	Moderna, Pfizer-BioNTech	648/12109 (5.35%)
Klugar et al.	Healthcare workers	599	Germany	39 *	§§	February–April 2021	Moderna, Pfizer-BioNTech, Astra Zeneca	21/599 (3.51%)
Lim et al.	Healthcare workers	1704	Singapore	NR	NR	February–April 2021	Pfizer-BioNTech	132/1704 (7.75%)
Oulee et al.	Healthcare workers	137	USA	NR	54.7	29 March–29 May 2021	Moderna, Pfizer-BioNTech	5/137 (3.64%)
Pourani et al.	Healthcare workers	761	Iran	28.08 (11.94)	70.3	June–July 2021	Astra Zeneca, Sinopharm, Sputnik, Bharat, Cuba-Pasteur, Pfizer-BioNTech, Moderna	95/761 (12.48%)
Riad et al. 1	Healthcare workers	92	Germany, Czech Republic	35.37 (12.62)	77.2	February–March 2021	Astra Zeneca	4/92 (4.34%)
Riad et al. 2	Healthcare workers	522	Slovakia	37.77 (11.61)	77	February–March 2021	Moderna, Pfizer-BioNTech, Astra Zeneca	18/522 (3.45%)
Riad et al. 3	General Population	539	Czech Republic	22.86 (2.05)	70.1	April–June 2021	Moderna, Pfizer-BioNTech	4/539 (0.74%)
Riad et al. 4	Healthcare workers	877	Czech Republic	42.56 (10.5)	88.5	27 January–27 February 2021	Pfizer-BioNTech	45/877 (5.13%)
Robinson et al.	Healthcare workers	33039	USA	NR	NR	December 2020–February 2021	Moderna, Pfizer-BioNTech	1541/33039 (4.66%)
Ruiz-Villaverde et al.	Healthcare workers	3969	Spain	46.4 (13.9)	73.1	27 December 20–1 September 2021	Pfizer-BioNTech	13/3969 (0.33%)

SD = Standard Deviation; NR = Not Reported; * Median (Interquartile range); ^ Age range; § BNT162b2 1st dose: 63.6, 2nd dose: 61.8; mRNA-1273 1st dose: 45.9, 2nd dose: 47.3; §§ mRNA vaccines: 73.6. Viral vector: 67.2.

## Data Availability

Data available as Supplementary Tables.

## Supplementary Materials

The following supporting information can be downloaded at: https://www.mdpi.com/article/10.3390/vaccines10091475/s1, Supplementary Figure S1. Risk of bias graph for each eligible study assessed by Johanna Briggs Institute (JBI) critical appraisal checklist for studies reporting prevalence data. Supplementary Figure S2. Risk of bias summary of each items assessed by Johanna Briggs Institute (JBI) critical appraisal checklist for studies reporting prevalence data. Supplementary Figure S3. Forest plot and pooled estimates of the incidence of cutaneous adverse reaction to SARS-CoV-2 vaccines in 22 eligible studies stratified by vaccine platform. Supplementary Figure S4. Forest plot and pooled estimates of the incidence of cutaneous adverse reaction to SARS-CoV-2 vaccines in 22 eligible studies stratified by study population. Supplementary Figure S5. Forest plot and pooled estimates of the incidence cutaneous adverse reaction to SARS-CoV-2 vaccine in 22 eligible studies stratified by study country. Supplementary Figure S6. Funnel plot for eligible studies assessing the incidence of cutaneous adverse reaction to SARS-CoV-2 vaccine in 22 eligible studies. Egger test *p* < 0.001. Supplementary Table S1. Search Queries. Supplementary Table S2. Characteristics of excluded studies. Supplementary Table S3. outcome measures of included studies.
