# Supplementary material for: Cutaneous Adverse Reactions to SARS-CoV-2 Vaccines: A Systematic Review and Meta-Analysis
Source: Vaccines (Basel). 2022 Sep 6;10(9):1475. doi: 10.3390/vaccines10091475 (PMC9504216; doi:10.3390/vaccines10091475)
Supplement: Supplementary file 1 [file vaccines-10-01475-s001.zip › Supplementary TABLEs.pdf]

Supplementary Table S1: Search Queries

Database	Search Strategy
PubMed / MEDLINE	("COVID-19 Vaccine*" OR "Sars-Cov-2 Vaccination*" OR "bnt162b2*" OR "Comirnaty*" OR "mrna-1273*" OR "Spikevax*" OR "ChAdOx1*" OR "Vaxzevria*" OR "AZD1222*" OR "ad26.cov2.s*") AND ("Skin*" OR "Hair*" OR "Cutaneous*" OR "dermatology*" OR "delayed*" OR "vesiculo*" OR "bullous*" OR "pustular*" OR "exanthematous*" OR "morbilliform*" OR "pernio-like*" OR "Purpuric*" OR "Annular*" OR "Lichenoid*" OR "Lymphomatoid*") AND ("reaction*" OR "effect*" OR "event*" OR "recurrence*" OR "reactivation*" OR "regression*" OR "Eruption*" OR "rash*" OR "flares*" OR "eczema*" OR "lesion*" OR "pemphigus*" OR "psoriasis*" OR "Erythema*" OR "Pityriasis*" OR "Necrolysis*" OR "pustulosis*" OR "itchiness*" OR "Lichen*" OR "Chilblain*" OR "vasculitis*" OR "alopecia*" OR "pernio*" OR "purpura*" OR "COVID toes*" OR "COVID arm*") NOT ("thrombocytopenia*" OR "cardiological*" OR "neurological*" OR "myocarditis*" OR "thromboembolic*" OR "myopathy*" OR "seroconversion*")
Web Of Science	(ALL=(COVID-19 Vaccine*) OR ALL=(Sars-Cov-2 Vaccination*) OR ALL=(bnt162b2*) OR ALL=(Comirnaty*) OR ALL=(mrna-1273*) OR ALL=(Spikevax*) OR ALL=(ChAdOx1*) OR ALL=(Vaxzevria*) OR ALL=(AZD1222*) OR ALL=(ad26.cov2.s*)) AND (ALL=(Skin*) OR ALL=(Hair*) OR ALL=(Cutaneous*) OR ALL=(dermatology*) OR ALL=(delayed*) OR ALL=(vesiculo*) OR ALL=(bullous*) OR ALL=(pustular*) OR ALL=(exanthematous*) OR ALL=(morbilliform*) OR ALL=(pernio-like*) OR ALL=(Purpuric*) OR ALL=(Annular*) OR ALL=(Lichenoid*) OR ALL=(Lymphomatoid*)) AND (ALL=(reaction*) OR ALL=(effect*) OR ALL=(event*) OR ALL=(recurrence*) OR ALL=(reactivation*) OR ALL=(regression*) OR ALL=(Eruption*) OR ALL=(rash*) OR ALL=(flares*) OR ALL=(eczema*) OR ALL=(lesion*) OR ALL=(pemphigus*) OR ALL=(psoriasis*) OR ALL=(Erythema*) OR ALL=(Pityriasis*) OR ALL=(Necrolysis*) OR ALL=(pustulosis*) OR ALL=(itchiness*) OR ALL=(Lichen*) OR ALL=(Chilblain*) OR ALL=(vasculitis*) OR ALL=(alopecia*) OR ALL=(pernio*) OR ALL=(purpura*) OR ALL=(covid toes*) OR ALL=(covid arm*)) NOT (ALL=(thrombocytopenia*) OR ALL=(cardiological*) OR ALL=(neurological*) OR ALL=(myocarditis*) OR ALL=(thromboembolic*) OR ALL=(myopathy*) OR ALL=(seroconversion*))

Supplementary Table S2: Characteristics of excluded studies

	PMID	Authors	Publication Year	Study design	Title	Reason for exclusion
1	33783873	Fernandez-Nieto et al.	2021	Cross-sectional	Skin manifestations of the BNT162b2 mRNA COVID-19 vaccine in healthcare workers. 'COVID-arm': a clinical and histological characterization	Only data about delayed local injection reaction are reported
2	33838206	McMahon et al.	2021	Registry-based study	Cutaneous reactions reported after Moderna and Pfizer COVID-19 vaccination: A registry-based study of 414 cases	No data about incidence of CARs are reported
3	33886411	Wang et al.	2021	Cross-sectional	Safety survey by clinical pharmacists on COVID-19 vaccination from a single center in China	No data about incidence of CARs are reported
4	34086881	Jacobson et al.	2021	Cross-sectional, survey-based	Incidence and Characteristics of Delayed Injection Site Reaction to the mRNA-1273 SARS-CoV2 Vaccine (Moderna) in a Cohort of Hospital Employees	Only data about delayed local injection reaction are reported
5	34254291	Català et al.	2022	Nationwide, multicentre, cross-sectional study	Cutaneous reactions after SARS-CoV-2 vaccination: a cross-sectional Spanish nationwide study of 405 cases	No data about incidence of CARs are reported
6	34288056	Papadimitriou et al.	2022	Cross-sectional study	Delayed localized hypersensitivity reactions to COVID-19 mRNA	No data about incidence of CARs are reported

					vaccines: a 6-month retrospective study	
7	34357839	Talamonti et al.	2021	Cross-sectional study	Safety of COVID-19 vaccines in patients with psoriasis undergoing therapy with anti-interleukin agents	No data about incidence of CARs are reported. Patients with psoriasis under anti-interleukin treatment is the study population, which differs from the target population of our study.
8	34362136	Skroza et al.	2021	Cohort study	Safety and Impact of Anti-COVID-19 Vaccines in Psoriatic Patients Treated with Biologics: A Real-Life Experience	No data about incidence of CARs are reported. Patients with psoriasis under anti-interleukin treatment are the study population, which differs from the target population of our study.
9	34517079	McMahon et al.	2022	Registry-based study	Clinical and pathologic correlation of cutaneous COVID-19 vaccine reactions including V-REPP: A registry-based study	No data about incidence of CARs are reported
10	34579248	Cugno et al.	2021	Cohort study	Increased Risk of Urticaria/Angioedema after BNT162b2 mRNA COVID-19 Vaccine in Health Care Workers Taking ACE Inhibitors	Only data about angioedema/urticaria are reported
11	34651054	Hibino et al.	2021	Cross-sectional study	Delayed Injection Site Reaction After mRNA-1273 Vaccination in Japan: A Retrospective, Cross-Sectional Study	Only data about delayed local injection reaction are reported
12	*34661934*	Rerknimitr et al.	2022	Cross-sectional, survey-based study	Cutaneous adverse reactions from 35,229 doses of Sinovavc and AstraZeneca COVID-19 vaccination: a	It is not clear whether reported findings refer to number of participants reporting CARs or number of CARs (also

					prospective cohort study in healthcare workers	multiple CARs per participant) for each administered dose.
13	34699117	Musumeci et al.	2022	Cohort study	Safety of SARS-CoV-2 vaccines in psoriatic patients treated with biologics: A real life experience	No data about incidence of CARs are reported. Patients with psoriasis under biologics treatment are the study population, which differs from the target population of our study.
14	34800601	Freeman et al.	2022	Registry-based study	Skin reactions to COVID-19 vaccines: An American Academy of Dermatology/International League of Dermatological Societies registry update on reaction location and COVID vaccine type	No data about incidence of CARs are reported
15	34837354	Burlando et al.	2022	Retrospective descriptive study	Cutaneous reactions to COVID-19 vaccine at the dermatology primary care	This study reports data on the incidence of vaccine-associated CARs in a group of patients referring to the dermatology clinic. This group differs from the target population of our study.
16	34866925	Wang et al.	2021	Cross-sectional, web survey-based	A Web-Based Survey on Factors for Unvaccination and Adverse Reactions of SARS-CoV-2 Vaccines in Chinese Patients with Psoriasis	This study reports data on the incidence of vaccine-associated CARs in a group of psoriatic patients. This group differs from the target population of our study
17	34871159	Patel et al.	2021	Cohort study	COVID-19 vaccine-related presumed allergic reactions and second dose administration by using a two-step graded protocol	This study reports data on the incidence of vaccine-associated CARs in a group of patients referring to the immunology and allergology clinic. This group differs from the target population of our study.

18	34897821	Cristaudo et al.	2022	Cohort study	Immunogenicity and safety of anti-SARS-CoV-2 BNT162b2 vaccine in psoriasis patients treated with biologic drugs	This study reports data on the incidence of vaccine-associated flares in a group of psoriatic patients. This group differs from the target population of our study. Furthermore, data about the general incidence of vaccine-associated CARs are not reported.
19	34927749	Massip et al.	2022	Cohort study	Cutaneous manifestations following COVID-19 vaccination: a multicentric descriptive cohort	Only data about patients who presented with vaccine-associated CARs are reported. No incidence of CARs in the general population is reported.
20	34983707	Magen et al.	2022	Cohort study	Chronic spontaneous urticaria after BNT162b2 mRNA (Pfizer-BioNTech) vaccination against SARS-CoV-2	No data about incidence of general CARs are reported
21	35004790	Huang et al.	2021	Cohort study	Exacerbation of Psoriasis Following COVID-19 Vaccination: Report From a Single Center	This study reports data on the incidence of vaccine-associated flares in a group of psoriatic patients. This group differs from the target population of our study. Furthermore, data about the general incidence of vaccine-associated CARs are not reported.
22	35067996	Potestio et al.	2022	Cross-sectional study	Atopic dermatitis exacerbation after COVID-19 vaccination in Dupilumab-treated patients	This study reports data on the incidence of vaccine-associated flares in a group of atopic dermatitis patients. This group differs from the target population of our study. Furthermore, data about the

						general incidence of vaccine-associated CARs are not reported.
23	35179254	Pakhchanian et al.	2022	Retrospective cohort study	Evaluating the safety and efficacy of COVID-19 vaccination in patients with hidradenitis suppurativa	This study reports data on vaccine safety in a group of patients with hidradenitis suppurativa. This group differs from the target population of our study.
24	35202509	Hamed Azzam et al.	2022	Cross-sectional study	COVID-19 vaccine in patients with dermal hyaluronic acid fillers in the tear trough: A retrospective study	This study reports data on vaccine safety in a group of patients who underwent dermal hyaluronic acid fillers procedures. This group differs from the target population of our study.
25	35243732	Özgen et al.	2022	Cohort study	COVID-19 severity and SARS-Cov-2 vaccine safety in pemphigus patients	This study reports data on vaccine safety in a group of pemphigus patients. This group differs from the target population of our study.
26	35335112	Hertel et al.	2022	Cohort study	Onset of Oral Lichenoid Lesions and Oral Lichen Planus Following COVID-19 Vaccination: A Retrospective Analysis of about 300,000 Vaccinated Patients	This study reports data only on the incidence of vaccine-associated Oral Lichen Planus and Oral Lichenoid Lesions.
27	35361536	Maruyama et al.	2022	Cross-sectional, survey-based study	Adverse reactions to the first and second doses of Pfizer-BioNTech COVID-19 vaccine among healthcare workers	The incidence of vaccine-associated CARs is reported for distinct age groups, but not for the whole study population. Data from this study cannot thus be pooled and analyzed with data from other studies.

28	35407429	Grieco et al.	2022	Cross-sectional study	Effects of Vaccination against COVID-19 in Chronic Spontaneous and Inducible Urticaria (CSU/CIU) Patients: A Monocentric Study	This study reports data on vaccine safety in a group of patients with chronic spontaneous urticaria. This group differs from the target population of our study.
29	35693768	Sprow et al.	2022	Chart review	Autoimmune Skin Disease Exacerbations Following COVID-19 Vaccination	This study reports data on vaccine safety in a group of patients with autoimmune skin diseases. This group differs from the target population of our study.
30	35062676	Rivera-Izquierdo et al.	2021	Cross-sectional study	Factors Associated with Adverse Reactions to BNT162b2 COVID-19 Vaccine in a Cohort of 3969 Hospital Workers	This article reports data from the same exact study reported by Ruiz-Villaverde et al. The study population, time frame, setting and incidence data regarding dermatological adverse reactions are exactly the same. However, this article could not be removed as a duplicate, since Rivera-Izquierdo et al. report data on systemic adverse reactions as well.
31	35723893	Potestio et al.	2022	Retrospective study	Reply to 'Cutaneous adverse effects of the available COVID-19 vaccines in India: A questionnaire-based study'. by Bawane J et al	This study reports data on the incidence of vaccine-associated CARs in a group of patients referring to the dermatology clinic. This group differs from the target population of our study.
32	35649530	Higashino et al.	2022	Retrospective Cross-sectional study	Assessment of Delayed Large Local Reactions After the First Dose of the SARS-CoV-2 mRNA-1273 Vaccine in Japan	The primary outcome of the study is to investigate the incidence of delayed large local reactions in vaccine

						recipients. Incidence of general cutaneous reactions is not reported.
33	35562372	Pinpathomrat et al.	2022	Case-control study	Immunogenicity and safety of an intradermal ChAdOx1 nCoV-19 boost in a healthy population	The study does not report the incidence of general cutaneous reactions.
34	35513961	Nantanee et al.	2022	Cohort study	Immunogenicity and reactogenicity after booster dose with AZD1222 via intradermal route among adult who had received CoronaVac	The study does not report the incidence of general cutaneous reactions.
35	35491497	Guarnieri et al.	2022		Adverse reactions to BNT162B2 vaccine in health care workers from an Italian Tertiary Care Hospital	The study does not report the incidence of general cutaneous reactions.
36	35758760	Tavakoli et al.	2022	Cohort study	Pediatric and adolescent COVID-19 vaccination side effects: A retrospective cohort study of the Iranian teenage group in 2021	The study population (pediatric-teenage vaccine recipients) differs from our target population (general adult population without underlying conditions).
37	Not Reported	Mohta et al.	2022	Prospective observational study	Clinical and histopathological correlation of the mucocutaneous COVID-19 vaccine reactions: a prospective observational study	The study does not report the incidence of general cutaneous reactions.
38	35470920	Hertel et al.	2022	Cohort study	Real-world evidence from over one million COVID-19 vaccinations is consistent with reactivation of the varicella-zoster virus	The study reports the incidence of VZV reactivation, not of general cutaneous reactions.
39	Not Reported	Al-Qazaz et al.	2022	Cross-sectional study	COVID-19 vaccination, do women suffer from more side effects than	The study reports the incidence of local reactions in women, not of general

					men? A retrospective cross-sectional study	cutaneous reactions in the general population.

Supplementary Table S3: outcome measures of included studies

PMID	Author	General incidence of CARs (%)	Incidence of CARs occurring after 1st dose (%)	Incidence of CARs occurring after 2nd dose (%)	Incidence of CARs occurring after both doses (%)	General incidence of CARs by vaccine type (%)	Type of cutaneous reactions, as reported by the included studies
34320413	Al Bahrani et al.	NR	307/1592 (19.28%)	NR	NR	NR	Skin rash (n = 307)
34976653	Almohaya et al.	73/3639 (2.00%)	73/3639 (2.00%)	26/1298 (2.00%)	NR	Pfizer – BioNTech: 73/3639 (2.00%)	Generalized rash (n = 73)
35429053	Bawane et al.	30/1029 (2.92%)	17/358 (4.75%)	13/671 (1.94%)	NR	NR	First dose (358 respondents):<br>- Swelling other than site of injection (n = 10)<br>- Urticaria within 24h (n = 2)<br>- Urticaria after 24h (n = 1)<br>- Pytiriasis rosea (n = 1)<br>- Morbilliform rash (n = 3)<br>Second dose (671 respondents):<br>- Swelling other than site of injection (n = 8)<br>- Urticaria after 24h (n = 1)<br>- Pytiriasis rosea (n = 2)<br>- Pernio/chilblain (n = 1)<br>- Urticarial vasculitis (n = 1)
35775860	Bostan et al.	2/234 (0.85%)	NR	NR	NR	NR	Exacerbation of psoriasis (n = 1)<br>Acute urticaria (n = 1)
35293657	Bukhari et al.	51/1021 (5.00%)	NR	NR	NR	NR	Maculopapular rash (n = 29)<br>Urticaria (n = 12)<br>Itching (n = 6)<br>Acne (n = 4)<br>Angioedema (n = 3)<br>Other skin manifestations (n = 8)
35780311	Cebeci Kahraman et al.	175/2189 (7.99%)	94/2290 (4.10%)	81/2189 (3.70%)	NR	First dose:<br>- CoronaVac: 60/2097 (2.9%)	First dose (2290 respondents):<br>- Herpes Zoster (n = 17)<br>- Pytiriasis rosea (n = 12)<br>- Petechial rash (n = 1)

						- BioNTech: 33/183 (18.0%)<br>Second dose:<br>- CoronaVac: 73/2060 (3.5%)<br>- BioNTech: 6/119 (5.0%)	- Dyshidrotic eczema (n = 4)<br>- Urticaria/Angioedema within 24h (n = 6)<br>- Urticaria/Angioedema after 24h (n = 15)<br>- Lichenoid reaction (n = 2)<br>- Erythromelalgia (n = 1)<br>- Contact dermatitis (n = 9)<br>- SDRIFE (n = 1)<br>- Disseminated herpes zoster (n = 1)<br>- Acneiform eruption (n = 1)<br>- Maculopapular rash (n = 9)<br>- Erythroderma (n = 1)<br>- Bullous pemphigoid (n = 2)<br>- Psoriasis vulgaris (n = 2)<br>Second dose (2189 respondents):<br>- Herpes Zoster (n = 10)<br>- Pytiriasis rosea (n = 10)<br>- Dyshidrotic eczema (n = 1)<br>- Urticaria/Angioedema within 24h (n = 6)<br>- Urticaria/Angioedema after 24h (n = 19)<br>- Lichenoid reaction (n = 2)<br>- Contact dermatitis (n = 8)<br>- Disseminated herpes simplex (n = 1)<br>- Maculopapular rash (n = 9)<br>- Chilblain (n = 2)<br>- Cutaneous vasculitis (n = 2)<br>- Psoriasis vulgaris (n = 3)<br>- Cutaneous sarcoidosis (n = 1)<br>- Linear IgA bullous dermatosis (n = 1)

35067984	Das et al.	50/4063 (1.23%)	NR	NR	NR	Covishield – Astra Zeneca: 50/4063 (1.23%)	Telogen effluvium (n = 18)<br>Wheals (n = 12)<br>Generalized pruritus without rash (n = 4)<br>Maculopapular rash (n = 4)<br>Pytiriasis rosea (n = 3)<br>Angio-edema (n = 3)<br>Eruptive pseudoangiomatosis (n = 2)<br>Lichen Planus (n = 2)<br>Non-blanchable petechial rash (n = 1)<br>Herpes simplex labialis (n = 1)<br>Pemphigus vulgaris (n = 1)<br>Cheiro-pedo pompholyx (n = 1)
34415637	Durmaz et al.	NR	33/221 (14.93%)	NR	NR	NR	First dose (221 respondents):<br>- Urticaria (n = 12)<br>- Herpes reactivation (Zoster: n = 2; Simplex: n = 2)<br>- Papulosquamous/Pytiriasiform lesions (n = 8)<br>- Angio-edema (n = 3)<br>- Type IV reaction [Erythema Multiforme, Lichenoid Drug Eruption, Drug Hypersensitivity Syndrome] (n = 3)<br>- Palmar erythema (n = 2)<br>- Small vessel vasculitis (n = 1)
34021625	Farinazzo et al.	28/19485 (0.14%)	NR	NR	NR	Pfizer – BioNTech: 28/19485 (0.14%)	Urticarial rash and diffuse urticaria (n = 9)<br>Erythema/dermatitis (n = 8)<br>Generalized pruritus (n = 3)<br>Swelling of face/eyelids (n = 3)<br>Herpes Zoster (n = 2)<br>Pytiriasis rosea-like rash (n = 2)<br>Fixed Drug Eruption (n = 1)<br>Cutaneous rash of the trunk (n = 1)<br>Morbilliform eruption (n = 1)

34622531	Grieco et al.	50/2740 (1.82%)	28/2740 (1.02%)	20/2740 (0.73%)	2/2740 (0.07%)	Pfizer – BioNTech: 30/2481 (1.21%)<br>Moderna: 4/37 (10.81%)<br>Astra Zeneca: 16/222 (7.21%)	Urticarial rash/Angioedema (n = 14)<br>Toxic erythema (n = 4)<br>Erythema multiforme (n = 3)<br>Pytiriasis rosea-like eruption (n = 3)<br>Stevens-Johnson-Syndrome (n = 1)<br>Morbilliform Drug Exanthema (n = 1)<br>Lymphomatoid drug reaction resembling PLEVA (n = 1)<br>Erythema nodosum (n = 1)<br>Late onset atopic dermatitis (n = 1)<br>Annular lichen planus (n = 1)<br>Pseudo chilblain relapsing with necrotic features at the second dose (n = 1)<br>Filler injection site reaction (n = 1)<br>Genital fixed drug reaction (n = 1)
34816647	Im et al.	93/2498 (3.72%)	43/1876 (2.29%)	50/1876 (2.67%)	NR	NR	First dose (1876 respondents)<br>- Urticaria (n = 43)<br>Second dose (1840 respondents)<br>- Urticaria (n = 50)
35058126	Kitagawa et al.	648/12109 (5.35%)	360/7291 (4.94%)	288/4818 (5.98%)	NR	NR	First dose (7291 respondents):<br>- Skin rash (n = 360)<br>Second dose (4818 respondents):<br>- Skin rash (n = 288)
34439984	Klugar et al.	21/599 (3.51%)	NR	NR	NR	Pfizer – BioNTech: NR<br>Moderna: NR<br>Astra Zeneca: 7/125 (5.60%)	Rash (n = 17)<br>Urticaria (n = 4)<br>Angioedema (n = 4)
34625758	Lim et al.	132/1704 (7.75%)	42/1704 (2.5%)	90/1704 (5.3%)	NR	NR	Skin reaction, not at the injection site (rash, hives, urticaria, itch)<br>- First dose (n = 42)<br>- Second dose (n = 90)

35028998	Oulee et al.	5/137 (3.64%)	2/137 (8.76%)	3/137 (5.84%)	4/137 (2.91%)	Pfizer – BioNTech: 7/62 (11.29%)<br>Moderna 17/75 (22.67%)	Dishydrotic Eczema Flare (n = NR)<br>Pernio/chilblain (n = NR)<br>Psoriasiform dermatitis (n = NR)
34820975	Pourani et al.	95/761 (12.48%)	NR	NR	8/262 (3.05%)	NR	Skin sensitivity (n = 40)<br>Exanthematous rash (n = 29)<br>Urticaria (n = 25)<br>Petechiae/purpura (n = 16)<br>Vesicular eruption (n = 8)<br>Pernio-like eruption (n = 8)<br>Angioedema (n =5)<br>Erythema Multiforme-like eruption (n = 2)<br>Zoster (n = 2)
34207369	Riad et al. 1	4/92 (4.34%)	NR	NR	NR	Astra Zeneca: 4/92 (4.34%)	Skin rash (n = 4)
34577573	Riad et al. 2	18/522 (3.45%)	NR	NR	NR	Pfizer – BioNTech: 18/522 (3.45%)	Rash (n = 8)<br>Angioedema (n = 13)
34681273	Riad et al. 3	4/539 (0.74%)	NR	NR	NR	NR	Skin rash (n = 2)<br>Skin eruption (n = 2)
33916020	Riad et al. 4	45/877 (5.13%)	NR	NR	NR	Pfizer – BioNTech: 45/877 (5.13%)	Rash (n = 28)<br>Urticaria (n = 10)<br>Other (n = 9)

34160555	Robinson et al.	1541/33039 (4.66%)	776/40640 (1.91%)	765/33939 (2.25%)	101/609 (16.58%)	Pfizer – BioNTech: 278/10445 (2.67%)<br>Moderna: 1263/30195 (4.18%)	First dose (40460 respondents):<br>- Itching/rash other than at the injection site (n = 599)<br>- Hives/urticaria (n = 162)<br>- Swelling/angioedema (n = 120)<br>Second dose (33939 respondents):<br>- Itching/rash other than at the injection site (n = 546)<br>- Hives/urticaria (n = 194)<br>- Swelling/angioedema (n = 125)<br>Both doses (609 respondents):<br>- Itching/rash other than at the injection site (n = 81)<br>- Hives/urticaria (n = 20)<br>- Swelling/angioedema (n = 16)
35775137	Ruiz-Villaverde et al.	13/3969 (0.33%)	NR	NR	NR	Pfizer – BioNTech: 13/3969 (0.33%)	Morbilliform rash (n = 10)<br>Herpes simplex reactivation (n = 5)<br>Herpes zoster reactivation (n = 1)<br>Distal hyperpigmentation (n = 1)<br>Generalized eczema (n = 1)

NR = Not Reported; SDRIFE = Simmetrical Drug-Related Intertriginous and Flexural Exanthema
