# Supplementary figures and images for: Cutaneous Adverse Reactions to SARS-CoV-2 Vaccines: A Systematic Review and Meta-Analysis
Source: Vaccines (Basel). 2022 Sep 6;10(9):1475. doi: 10.3390/vaccines10091475 (PMC9504216; doi:10.3390/vaccines10091475)

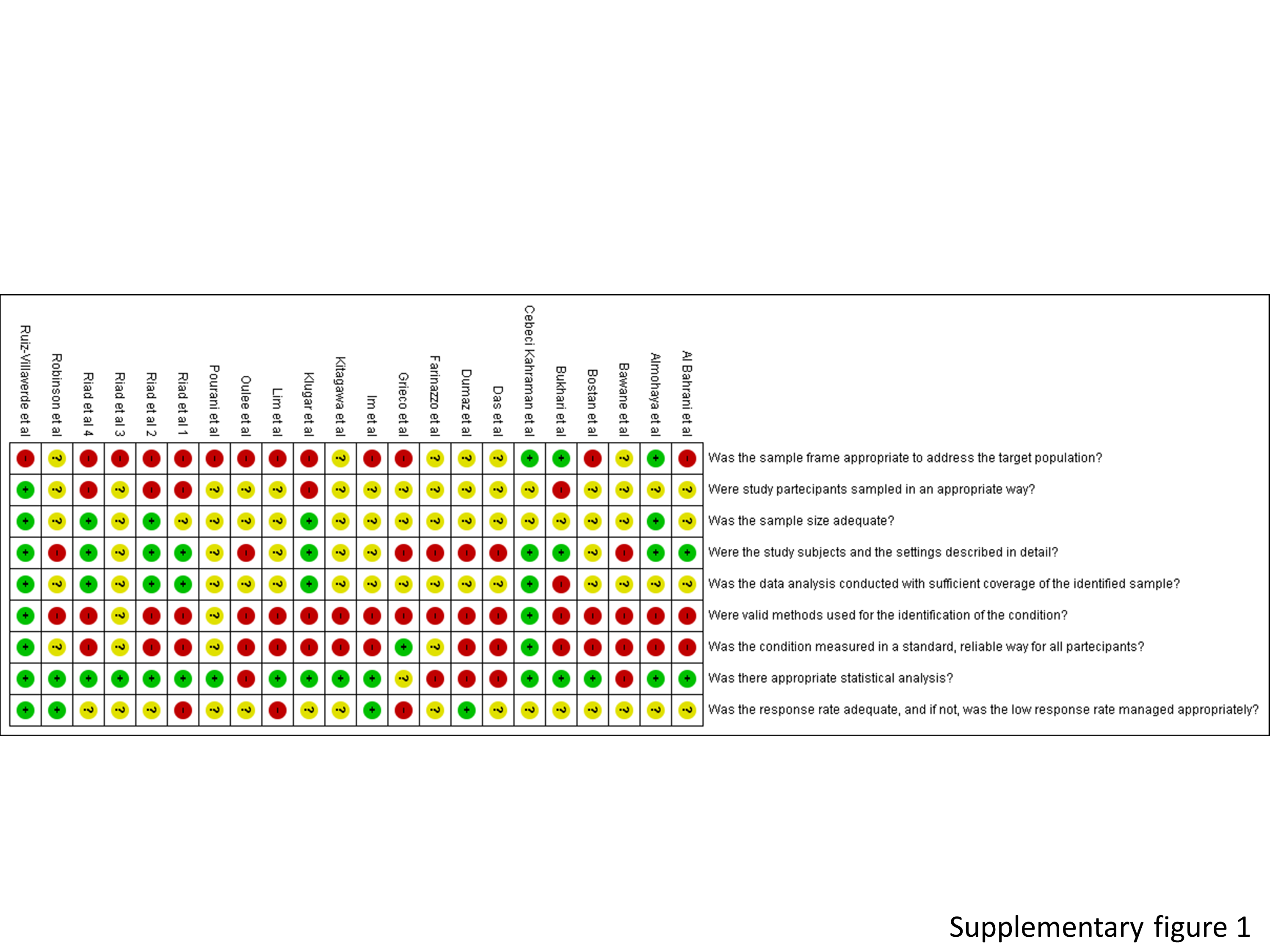

Supplement: Supplementary file 1 [file vaccines-10-01475-s001.zip › Supplementary figure S1.TIF]

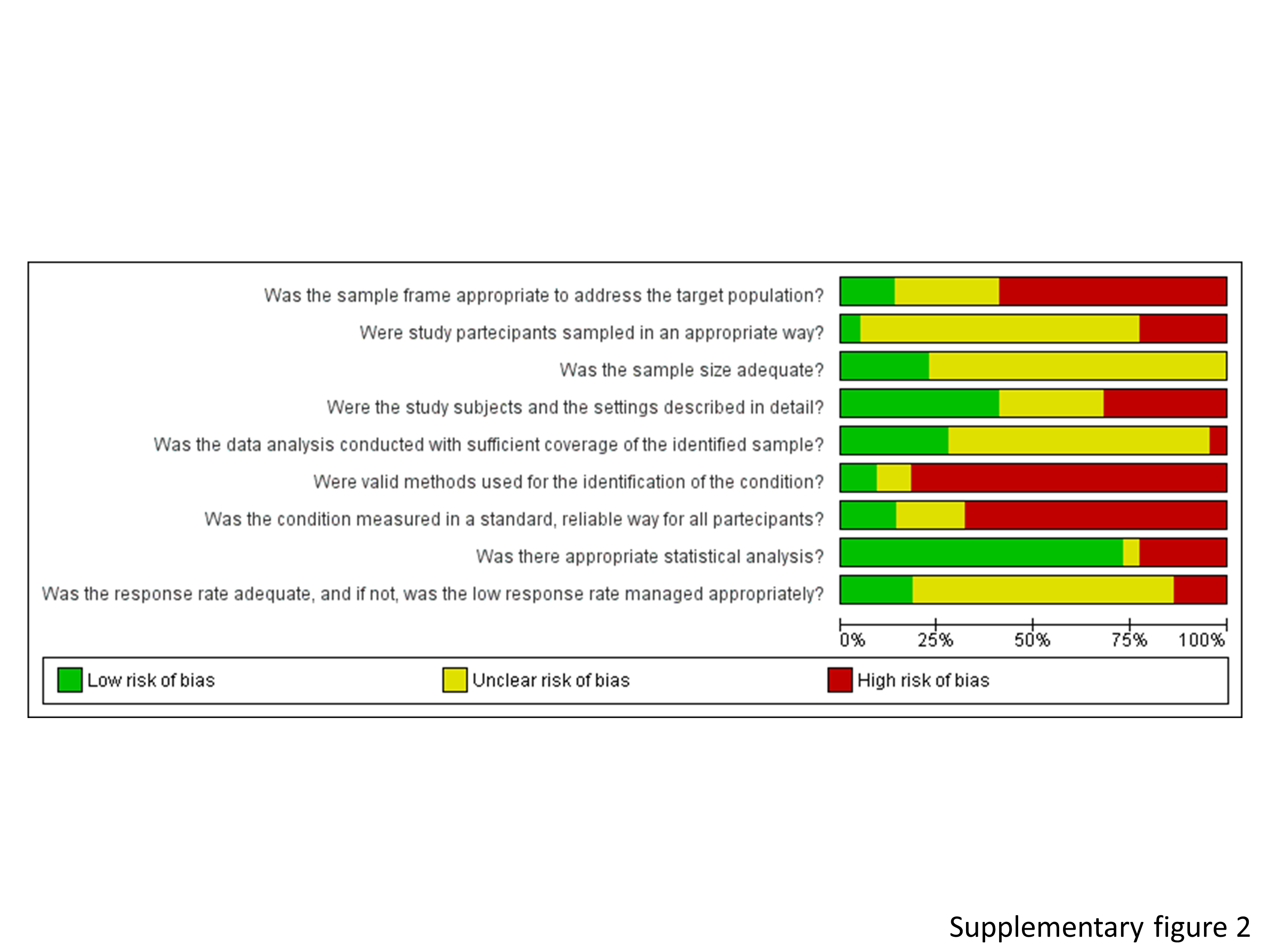

Supplement: Supplementary file 1 [file vaccines-10-01475-s001.zip › Supplementary figure S2.TIF]

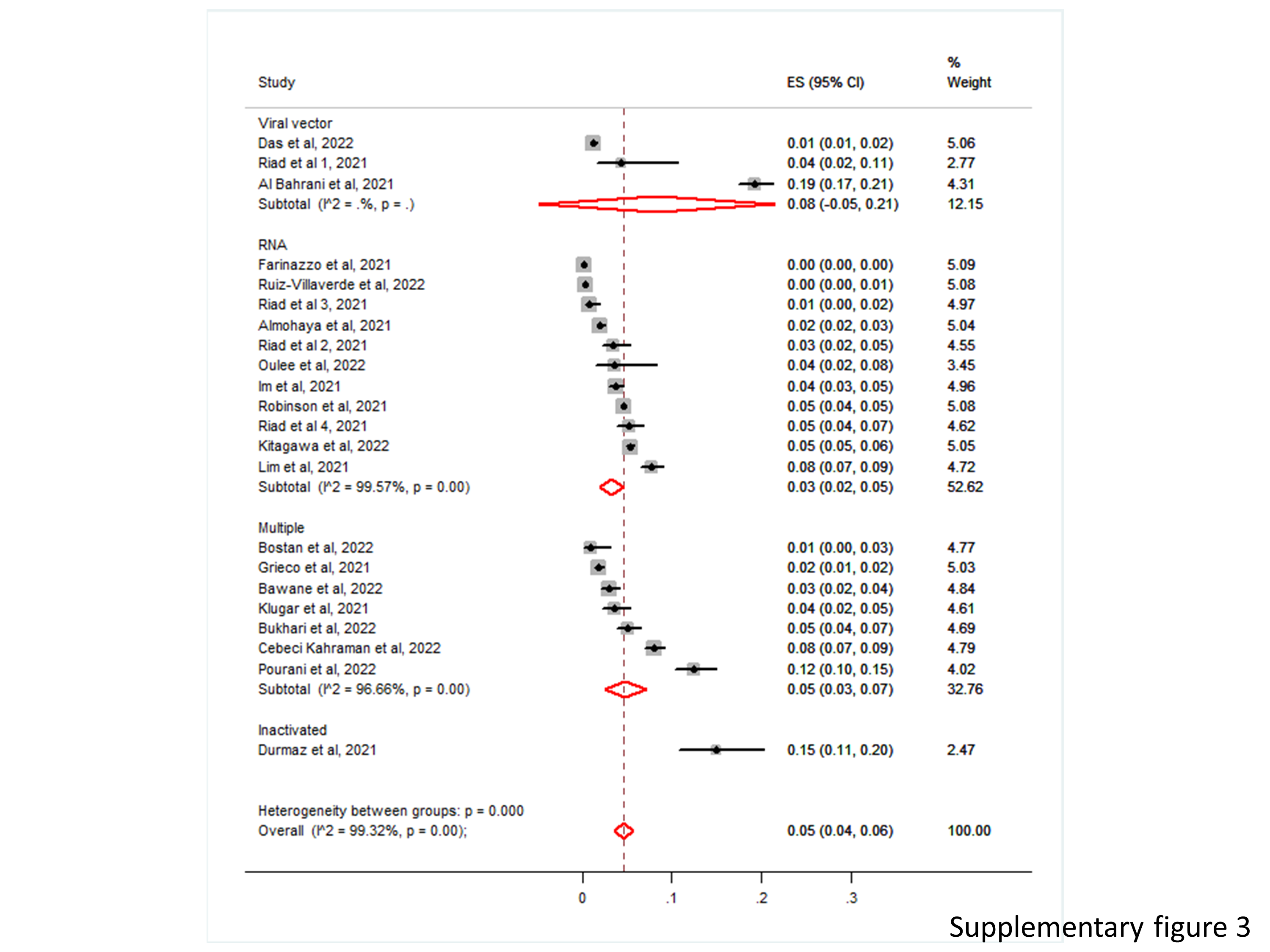

Supplement: Supplementary file 1 [file vaccines-10-01475-s001.zip › Supplementary figure S3.TIF]

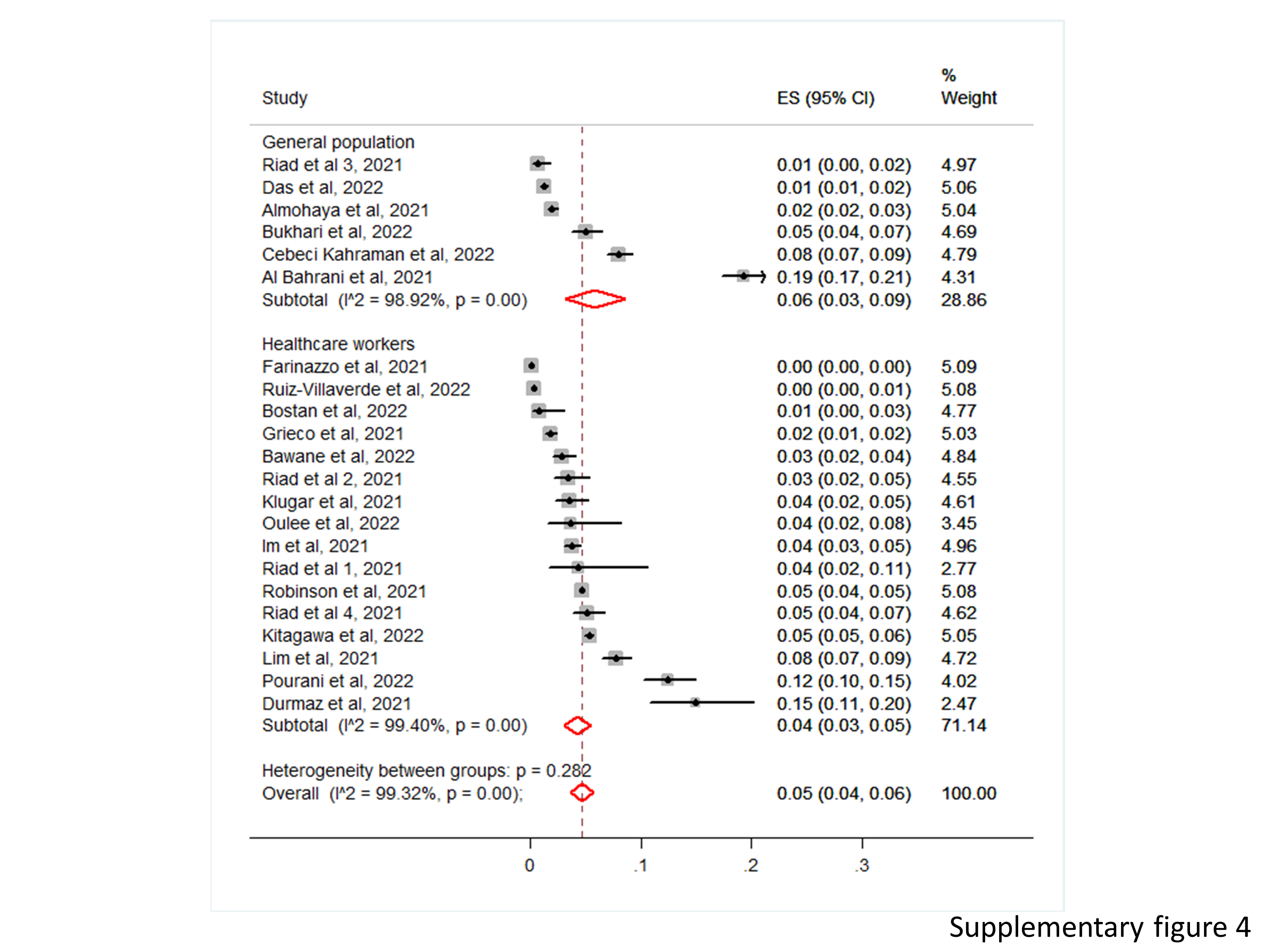

Supplement: Supplementary file 1 [file vaccines-10-01475-s001.zip › Supplementary figure S4.TIF]

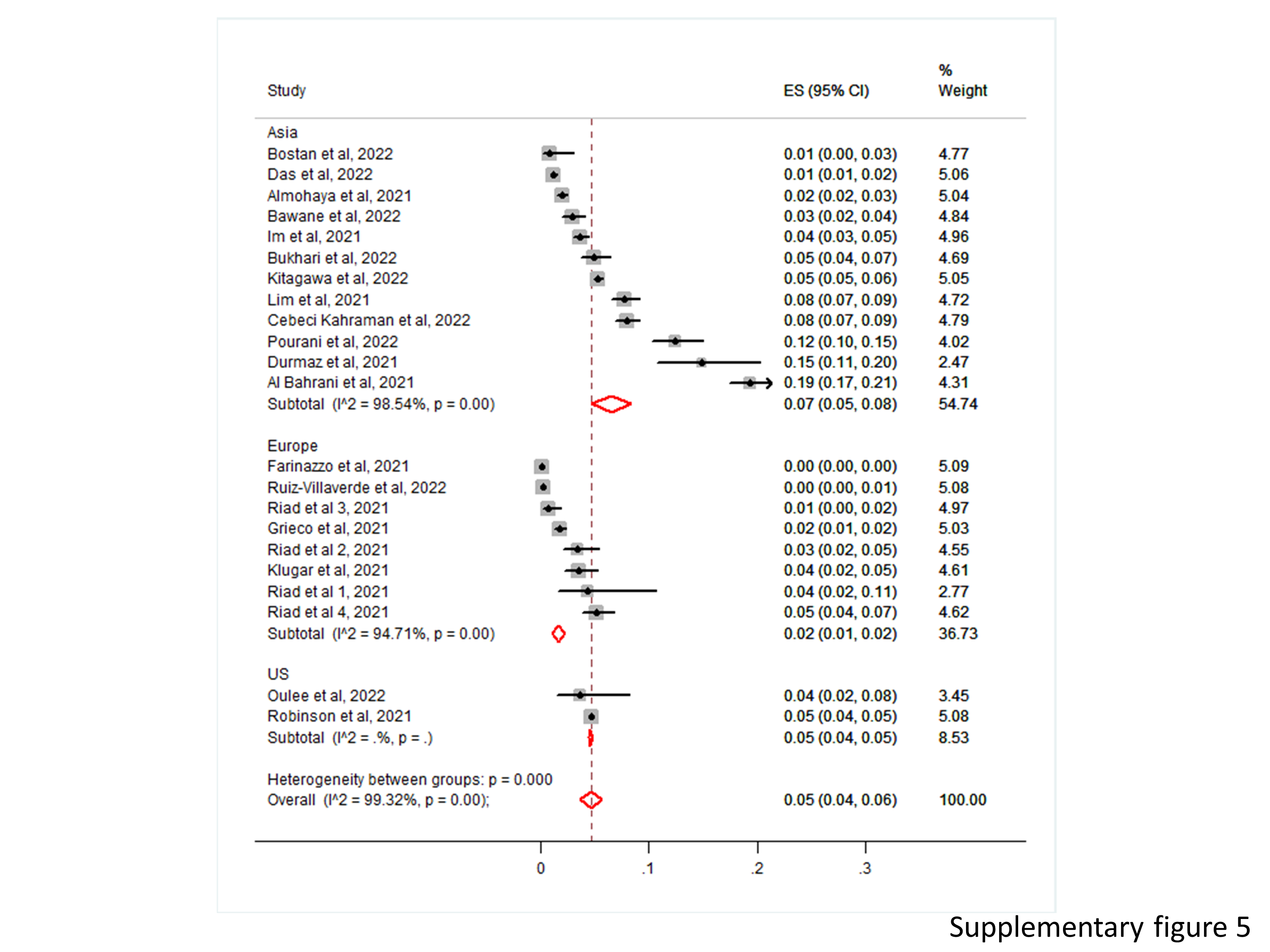

Supplement: Supplementary file 1 [file vaccines-10-01475-s001.zip › Supplementary figure S5.TIF]

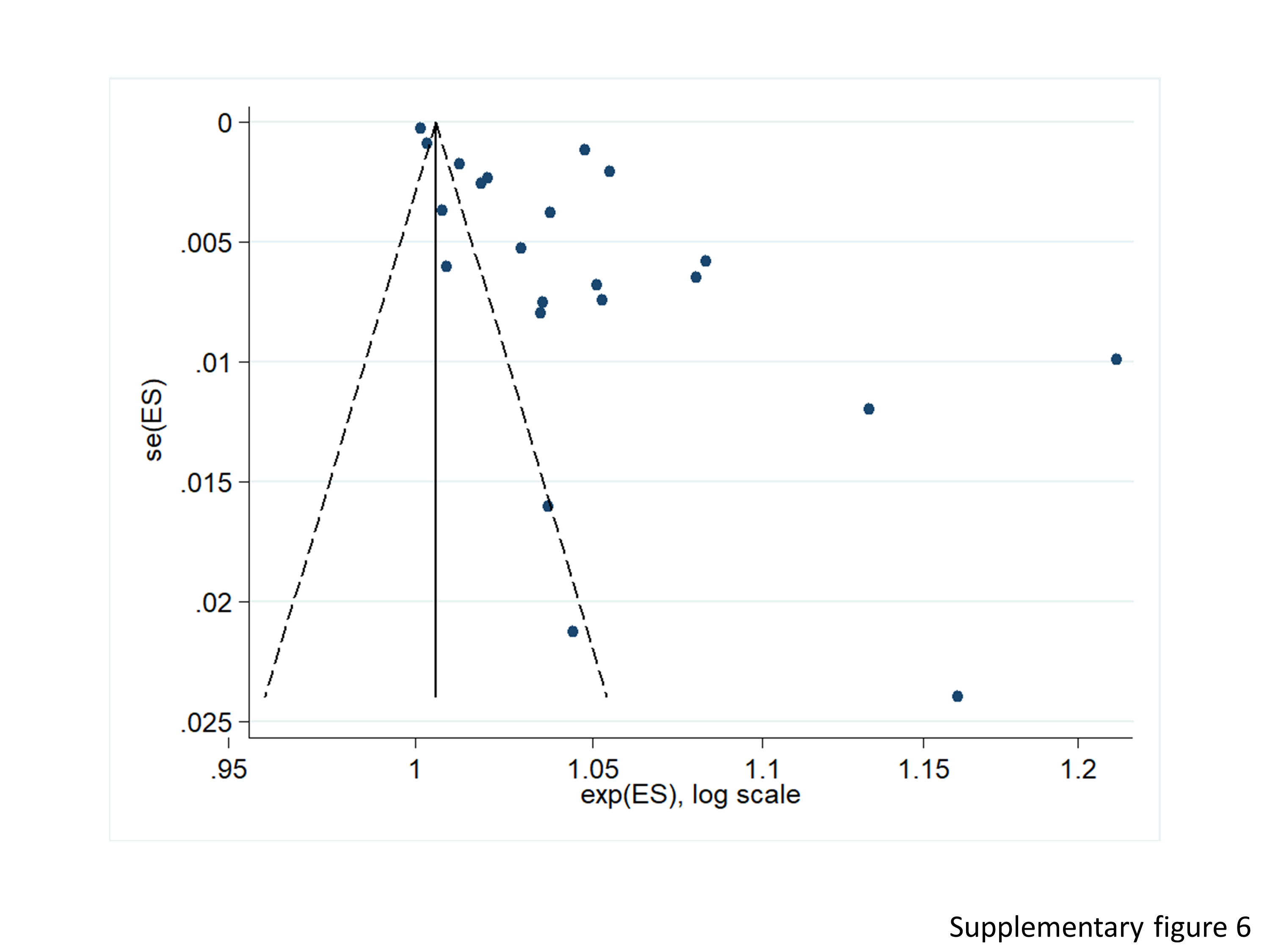

Supplement: Supplementary file 1 [file vaccines-10-01475-s001.zip › Supplementary figure S6.TIF]
